# Family Science Capital Factors Affecting Early Childhood Science Learning: A Systematic Review

**DOI:** 10.3390/bs15091152

**Published:** 2025-08-25

**Authors:** Bingying Gu, Dongqing Yu, Meimei Liu

**Affiliations:** 1 Faculty of Education, Northeast Normal University, Renmin Street, Changchun 130024, China; guby614@nenu.edu.cn; 2 Faculty of Behavioural and Movement Sciences, Vrije Universiteit Amsterdam, 1081 HV Amsterdam, The Netherlands; m.m.liu@vu.nl; 3 Research Institute Learn, 1081 HV Amsterdam, The Netherlands

**Keywords:** family science capacity, early childhood, science learning, systematic review

## Abstract

This study investigated how family science capital—comprising cultural, practice, and social dimensions—shapes early childhood science learning. Despite growing interest in informal science education, prior research has often overlooked the complex, interactive, and context-dependent nature of family science capital. Using a four-dimensional learning framework and the PRISMA method, this review synthesized findings from 56 studies to show that family science capital plays a central role in children’s early science learning. It emphasizes the need to understand how different capital configurations shape outcomes and calls for targeted policy efforts to support disadvantaged families. Strengthening family science capital through collaborative, cross-sectoral strategies is essential for promoting equity and improving early science education.

## 1. Introduction

In recent years, family science capital has emerged as a prominent theme in global science education research. The U.S. National Science Foundation’s Advancing Informal STEM Learning (AISL) initiative has supported the strengthening of family science capital through resource provision, parent training, and the enhancement of social capital, effectively promoting capital transmission (Raynal et al., 2021). The accumulation and intergenerational transfer of family science capital represent a critical factor in promoting children’s science learning (Archer et al., 2015; Suortti et al., 2023; Young et al., 2024). Studies have shown that families with high levels of science capital place greater emphasis on the societal value of science, more frequently use science websites and visit informal science learning venues, and exhibit stronger science identity, thereby offering better support for their children’s scientific development (Suortti et al., 2023). Further analyses indicate that the influence of family science capital manifests significantly during early childhood. By age three, it can predict science literacy development over the following four years (Bae et al., 2023), laying a foundation for participation in K-12 science learning, fostering scientific identity, and potentially mitigating the negative impact of gender stereotypes, thereby narrowing participation gaps in science (Leech, 2024).

Science learning serves as a vehicle for enhancing individuals’ understanding of science, constructing knowledge, and fostering scientific literacy and responsible citizenship (Holbrook & Rannikmae, 2007). It encompasses multidimensional development. This study adopted a four-dimensional framework to elucidate how family science capital supports children’s science learning, grounded in the kindergarten-oriented framework proposed in Taking Science to School (Philip Bell et al., 2009; Shouse et al., 2007). This framework comprises knowledge, which includes understanding scientific concepts and using scientific tools such as magnifiers and measuring instruments; process, which involves scientific inquiry skills, reasoning, reflection, collaboration, and documentation; attitude, which captures curiosity, interest, persistence, and self-efficacy; and the socio’cultural dimension, which emphasizes the construction of a “scientific community” through interactions among family, school, and community, encompassing science identity and STEM identity (Mantzicopoulos et al., 2013; Young et al., 2024).

Building on the above, the accumulation and intergenerational transmission of family science capital emerges as a key theoretical framework for understanding disparities in children’s science learning (Archer et al., 2015; Young et al., 2024). Understanding the specific elements of family science capital that affect young children’s science learning is of paramount importance. Although increasing attention has been paid to parental engagement in informal science education, significant theoretical gaps remain regarding the mechanisms by which family science capital influences early childhood science learning.

Firstly, the limited perspectives adopted by existing studies have impeded the systematic deconstruction of the components of family science capital. While some studies have focused on the behavioral types of parental engagement in informal STEM activities and their effects on school readiness (Sheehan et al., 2018), they have often overlooked the diverse elements of science capital embedded in the family setting. Other studies have considered parental STEM beliefs and self-efficacy, yet their analytical frameworks remain confined to individual psychological levels, failing to conceptualize the family as a cultural conduit for science capital transmission (Alexandre et al., 2022). Secondly, existing research tends to isolate specific dimensions, with some focusing solely on the predictive role of individual variables such as scientific career aspirations (Kontkanen et al., 2024), lacking an integrated analysis of how various elements of family science capital interact synergistically. This fragmented research paradigm hinders a holistic understanding of family science capital as a structured resource. Moreover, there exists a clear contextual imbalance in the literature, with reviews predominantly concentrating on informal learning environments such as museums and science centers, examining their effects on science skills, mathematics proficiency, and socio-emotional development (Alexandre et al., 2022; Sheehan et al., 2018). In contrast, the potential moderating effects of family science capital within formal educational settings and across learning contexts remain underexplored. This contextual segmentation restricts a comprehensive understanding of the overarching influence of family science capital.

To address the above limitations, this study draws on existing definitions of science capital, defining family science capital as the collection of all science-related knowledge, attitudes, experiences, and resources possessed by individuals (Archer et al., 2015; Leech, 2024; Suortti et al., 2023). Specifically, family science capital is conceptualized as the multidimensional interactions between family members and other actors, leveraging diverse science-related experiences and resources in both formal and informal contexts to enhance children’s advantages in science learning (Sheehan et al., 2018). This includes all science-related knowledge, attitudes, experiences, and resources within the family—not limited to biological parents but also encompassing any adults assuming caregiving roles, such as grandparents and legal guardians—and their interactions with other participants in these contexts. Family science capital integrates cultural, practice, and social capital, comprising the following dimensions: (1) scientific cultural capital, which includes scientific literacy, dispositions and preferences toward science, and engagement with the science labor market; (2) practice capital, which includes science-related media consumption, participation in out-of-school science activities, and engaging children in science-related conversations; and (3) social capital, which includes family members’ scientific credentials and social networks. This study focuses on examining the elements of family culture, practice, and social capital that influence early childhood science learning. Accordingly, we applied the PRISMA methodology to conduct a systematic review of recently published peer-reviewed studies describing these projects. To ensure the systematicity and transparency of the literature review, we rigorously adhered to the Preferred Reporting Items for Systematic Reviews and Meta-Analyses (PRISMA) guidelines. From the perspectives of cultural, practice, and social family capital, this study identifies the key elements influencing early childhood science learning and explores how they exert such influence. Based on this framework, this study addresses the following four research questions:

(1) What are the descriptive characteristics (e.g., publication year, location, research methods, and research topics) of the included studies?
(2) What are the key elements of family cultural science capital that influence early childhood science learning, and how do they exert this influence?
(3) What are the key elements of family practice science capital that influence early childhood science learning, and how do they exert this influence?
(4) What are the key elements of family social science capital that influence early childhood science learning, and how do they exert this influence?

## 2. Methods

This study constitutes a systematic review focusing on elements of family science capital that influence early childhood science learning. To ensure a rigorous and comprehensive synthesis of relevant studies, the researchers adhered to the procedures outlined in the Preferred Reporting Items for Systematic Reviews and Meta-Analyses (PRISMA) framework. Similar to general qualitative methodologies, the core components of this process included the extraction, comparison, and contrast of concepts or themes and the synthesis of findings into a conceptual taxonomy. Specifically, to enable a robust explanation and description of the phenomenon under study, we strictly adhered to the detailed procedures outlined in PRISMA. Beginning with the formulation of research questions, we applied predefined inclusion criteria to conduct systematic literature searches and study identification, followed by data screening, extraction, and analysis (Appendix A). Below, we briefly outline each methodological step, along with the inclusion and exclusion criteria that guided this study.

### 2.1. Inclusion and Exclusion Criteria

Given the aim of this review, several criteria were established to guide the inclusion of studies. First, regarding the study population, this review included research on the influence of family science capital on early childhood science learning, limited to studies on preschool-aged children or examined experiences between ages 0 and 8. Second, studies were included only if they were published after 2007, as the publication of Taking Science to School in that year marked the establishment of a developmentally appropriate science learning framework for kindergarten-aged children, thereby representing a turning point in the scholarly trajectory of science learning research. Third, studies had to be published in English, in line with the linguistic competencies of the research team. Fourth, studies were required to focus on elements of family science capital that relate to at least one aspect of children’s science learning. The principal outcomes of interest were the specific elements of family science capital identified as having an impact on children’s science learning. Fifth, to ensure the quality and validity of the review, the search was confined to peer-reviewed articles published in academic databases.

### 2.2. Literature Search

The literature search was conducted using the PRISMA framework across four major databases: ERIC, PsycINFO, Web of Science, and Scopus. Supplementary tools such as Google Scholar and ProQuest were also utilized. To ensure reproducibility, search results were systematically recorded in an online-accessible spreadsheet after duplicate entries had been removed.

**Figure 1:**
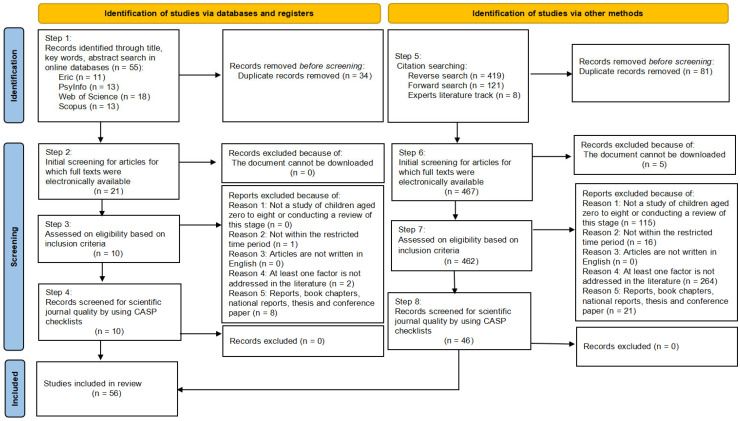
Preferred Reporting Items for Systematic Review flowchart.

The initial database search was conducted using English-language keywords structured via Boolean logic. The search combined terms representing science capital (e.g., “science capital”), early science learning (e.g., “early science learning”), home environment (e.g., “home environment”), and early childhood (e.g., “early childhood”). Boolean operators were applied across all fields to identify the relevant literature (see Appendix B). After removing duplicate records, outdated publications, studies that did not match the subject criteria, and non-journal articles, a total of ten studies met the initial eligibility criteria (see Figure 1).

### 2.3. Screening Strategy

Given the limited number of studies identified in the initial search, a snowball sampling strategy was employed to enhance the comprehensiveness of the review. This included backward citation tracking of references from the initially selected studies, forward citation tracking via Google Scholar to identify subsequent studies that cited the initial publications, and expert citation mapping, which involved collecting the complete list of publications by prolific authors in the field, such as the team led by Louise Archer. All studies retrieved through these supplementary methods were re-evaluated against the established inclusion criteria as detailed in the PRISMA flow diagram.

The next stage involved screening article abstracts to determine whether they satisfied the predefined inclusion and exclusion criteria. The first and second authors independently reviewed all abstracts to assess eligibility, considering factors such as topical relevance, clarity of English expression, and quality factors such as methodological rigor, relevance of research questions, and reasonableness of conclusions. Where abstracts lacked sufficient information, the full texts were consulted to decide whether to include or exclude the study. Five studies were removed due to unavailability online. For studies deemed preliminarily eligible, full texts were obtained and assessed. All 56 included studies were then subjected to an additional round of independent review by the two researchers. During this step, the Critical Appraisal Skills Programme (CASP) was used to evaluate the quality of each study, focusing on the alignment between research aims, methods employed, and reported results. Discrepancies or concerns raised during this process were discussed and resolved through consensus between the two researchers.

### 2.4. Data Analysis

In the data analysis phase, descriptive information was systematically collected for each study, including publication year, methodological approach (quantitative, qualitative, or mixed methods), country of origin, research aims, and findings. This information was documented in a standardized Excel template. Appendix A presents selected characteristics of the included studies based on extracted data such as author, year, country, and methodology.

To determine thematic focus and gain a deeper understanding of each publication, the review adopted an inductive coding approach for thematic analysis, as described by Terry et al. (2017). Each study’s aims and content were analyzed in depth. The coding structure emerged from both theoretical insights and empirical observations drawn from the preliminary review of the studies. To ensure thematic saturation and enhance the validity of the classification framework, the first author initially coded the first half (28) of the included studies, generating a provisional coding taxonomy. The second author then applied and refined this coding structure to the remaining studies, incorporating modifications as deemed relevant. The final taxonomy was developed through dialogic synthesis between the two authors, which involved comparing levels of analytical abstraction and carefully integrating theoretical nodes.

To provide a clear account of the thematic analysis process and results and to enhance analytical transparency, we constructed a hierarchical theme–category–code framework (Appendix C) and provided concrete examples of the coding process (Appendix D). The final analysis identified three core themes—family cultural science capital, family practice science capital, and family social science capital—along with their subcategories (subthemes) and representative initial codes. Each theme, category, and key code was accompanied by an operational definition. Appendix C illustrates how data segments were extracted from the original studies, assigned initial codes, and grouped into corresponding categories and themes. This structured framework ensured a high degree of consistency between the analytical outcomes and the study’s findings.

## 3. Results

### 3.1. Descriptive Statistics

**Figure 2:**
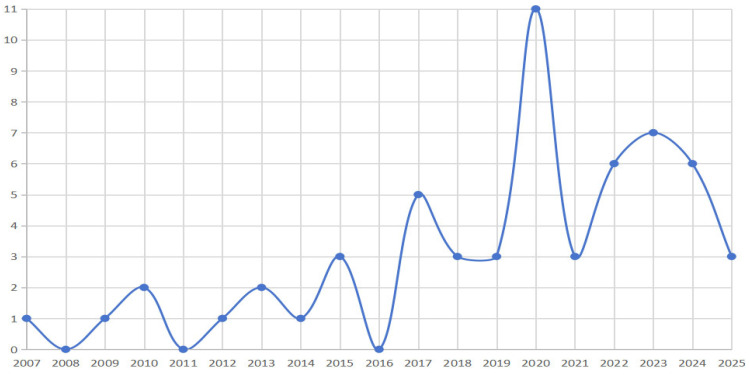
Yearly publication volume: 2007–2025.

This review systematically tracked 56 studies published between 2007 and 2025 and found a marked asymmetrical growth pattern in the field (Figure 2). Specifically, from 2007 to 2019, the field experienced a slow accumulation phase, with a total of 22 publications, averaging 1.69 per year, characterized by an ‘intermittent–emergent’ pattern, including years with no publications (2008, 2011, 2016). In contrast, the period from 2020 to 2025 represented a phase of rapid development, with an average annual output of 5.67 studies—an increase of 235.50% compared to the earlier phase—indicating sustained research momentum. Notably, 2020 marked a peak with 11 publications, more than doubling the previous year’s output, which may be attributed to the COVID-19 pandemic driving families to become central settings for science education.

In terms of geographic distribution, research on the impact of family science capital on early science learning showed marked spatial clustering, with a strong concentration in North America. Excluding review articles, data analysis revealed that among the 50 empirical studies, the United States overwhelmingly dominated the field, contributing 42 studies, accounting for 84% of the total sample. Although a broader range of European countries was represented, their overall contributions were limited, with five countries—the United Kingdom (1), Germany (2), Spain (2), Finland (1), and the Netherlands (1)—collectively accounting for only 14%. Asia was represented solely by a single study from Turkey (2%). This geographic imbalance likely reflects the developmental trajectory of STEM education systems: as the birthplace of STEM education reforms, North America holds an inherent advantage in the accumulation of research in science-related fields, whereas scholarly engagement in other regions has remained comparatively limited.

A panoramic methodological scan revealed the dominance of quantitative research among the 50 empirical studies (six review studies). Quantitative designs accounted for 72% (n = 36) of empirical studies and featured considerable heterogeneity in sample sizes, ranging from 44 to 15,847 participants. These studies utilized both cross-sectional and longitudinal designs and applied diverse analytic strategies, including correlation analyses, exploratory and confirmatory factor analyses, multiple regression, logistic regression, and structural equation modeling. In contrast, qualitative research made up 18% (n = 9) and emphasized interpretive depth, typically analyzing data obtained through interviews, transcripts from group sessions or workshops, verbal feedback, and ethnographic field recordings. These qualitative studies often included visual materials, such as video diaries and photo documentation, to support findings on family science capital and early learning. Mixed-methods studies (n = 5, 10%) offered an integrated lens, combining the explanatory strengths of qualitative inquiry with the generalizability of quantitative data. These studies typically used qualitative narratives to contextualize survey findings, achieving a multidimensional understanding of the phenomena under investigation.

Notably, analysis of the 56 included studies revealed a pronounced thematic imbalance, with family science practice capital emerging as the dominant focus. Specifically, 38 studies (67.86%) concentrated on practice capital, followed by 14 studies (25.00%) addressing family science social capital, and only four studies (7.14%) examining cultural capital. This indicates that existing research has been heavily oriented toward practice capital, with comparatively limited attention to social and cultural capital, highlighting an uneven distribution of research themes in the field. Subsequent sections provide a detailed, dimension-specific examination of these elements and their mechanisms of influence.

### 3.2. Family Cultural Capital and Early Childhood Science Learning

Family cultural capital, which includes the knowledge, attitudes, and experiences of family members, is significantly associated with early science learning outcomes (Kontkanen et al., 2024). Four core studies identified in this review focus on two dimensions: family science literacy (one study) and science dispositions and preferences (three studies). The integrated analysis indicates that family cultural capital plays a critical role in process guidance and interest stimulation through pathways such as knowledge representation and the construction of a supportive family ecology.

Family science literacy refers to the degree to which family members possess scientific knowledge, methods, and principles, primarily influencing children’s science learning through the accurate representation of knowledge. One reviewed study (Eberbach & Crowley, 2017) examined this in an experimental setting, showing how parents’ scientific knowledge shaped their children’s observational capacities. Seventy-nine parent-child dyads were grouped into high and low science knowledge conditions and observed pollen transfer phenomena in a botanical garden. Parents with higher science literacy were more likely to use disciplinary terminology (e.g., “stamen,” “pollen transfer”) and helped children form structure-function associations (e.g., “bee leg hairs capture pollen”). Path analysis showed that parents’ disciplinary talk significantly predicted children’s learning outcomes in observation tasks (β = 0.33, *p* < 0.001), suggesting that scientific literacy enhances observation depth through more precise knowledge representation.

Science dispositions and preferences reflect the perceived value and emotional orientation toward science, shaping their attitudes and levels of importance placed on science. These dispositions primarily influence children’s science attitudes through value transmission and emotional resonance. Three reviewed studies confirmed that such dispositions, embedded in the dynamic co-evolutionary relationships within families, enhance children’s science learning attitudes. A combined analysis further demonstrated a significant positive correlation between parental attitudes toward science and children’s science interest (r = 0.41, *p* < 0.01) and science self-concept (Kontkanen et al., 2024; Salvatierra & Cabello, 2022). Additionally, one study, grounded in family systems theory, conducted in-depth tracking of 15 families participating in the ‘Launching Engineering’ project, revealing the sustained nature of children’s interest development. The study highlighted how parental beliefs and children’s interests form a dynamic co-evolving system within families, operating through three pathways—providing learning opportunities, shaping interaction patterns, and transmitting cultural values—collectively extending children’s trajectories in science learning (Pattison et al., 2020).

### 3.3. Family Science Practice Capital and Early Childhood Science Learning

Family science practice capital, as a core component of family science capital, refers to the science-related behaviors and practices of family members. This review systematically examined 38 cutting-edge studies, focusing on three major dimensions of practice: science media consumption (two studies), participation in out-of-school science activities (11 studies), and family science dialogue (25 studies). The findings revealed that family science practices play a multidimensional and dynamic role in children’s cognitive development.

Family science media consumption refers to the frequency and intensity with which family members access science-related content through media channels such as television and the internet. This form of media engagement influences children’s science identity and skills through virtual–real-world connections, with its effects moderated by patterns of media use. In the context of rapid digital education development, it demonstrates unique value. A large-scale longitudinal study (n = 15,847) found that college students who had engaged in one hour of weekly family-based science media interaction during childhood demonstrated a 0.51 standard deviation increase in long-term STEM identity, an effect that remained significant after controlling for parental education and family support (Dou et al., 2019). Notably, the short-term educational effectiveness of science media may be moderated by parental occupation. One study found that in non-STEM occupational households, an additional hour of weekly science program viewing among preschoolers was associated with a 0.16 standard deviation fluctuation in math skills and a 0.14 standard deviation fluctuation in science skills (β = −0.27, *p* = 0.001) (Sheehan et al., 2018). These findings underscore the double-edged nature of science media, suggesting that its educational value is maximized when appropriate usage norms are established within the family.

Participation in out-of-school science activities refers to the degree of engagement in informal science learning environments. Such participation promotes knowledge integration and the internalization of interest through immersion in diverse learning contexts (Paños & Ruiz-Gallardo, 2020; Polinsky et al., 2023). First, knowledge interactions within out-of-school science activities can deepen children’s understanding. Studies employing mediation models have shown that home-based science learning environments significantly enhance science literacy among five-year-old children. For instance, a unit increase in the frequency of family science activities was associated with an 18% improvement in children’s science knowledge (β = 0.18), even after controlling for general cognitive stimulation (Bae et al., 2023; Junge et al., 2021). This suggests a positive correlation between family support for science and children’s science learning (Dominke et al., 2025). Interestingly, a distinction has been made between dialogic interaction capital (DIC) and science and engineering practice (SEP) capital. DIC refers to family dialogues or knowledge-sharing activities focused on core concepts across domains such as life sciences, earth and space sciences, physical sciences, and engineering and technology applications. SEP refers to parent-guided methods for engaging children in scientific inquiry and constructing scientific knowledge. Findings showed that DIC frequency significantly predicted children’s core science knowledge (β = 0.17, *p* < 0.05), whereas SEP frequency, while considered critical for deepening scientific understanding, did not reach significance (β = 0.13, *p* = 0.117) (Westerberg et al., 2022).

Furthermore, longitudinal studies of museum-based educational programs found that children aged 6–8 who participated in family engineering design projects demonstrated significantly enhanced metacognitive skills, with greater narrative coherence and recall detail than the control group (Pagano et al., 2020). Practice-based science learning also complements family storytelling, particularly in culturally diverse families. A case study involving Latinx families showed that combining oral storytelling with book reading facilitated children’s understanding of scientific inquiry and abstract concepts (Haden et al., 2023). Additionally, sustained participation fosters cumulative interest. Longitudinal research has found a significant positive correlation between the frequency of family science activity participation and the persistence of children’s interest (r = 0.42, *p* < 0.01). A longitudinal study of four-year-olds from low-income families confirmed that structured engagement with science books, museum visits, and self-directed explorations effectively nurtured science interest (Pattison & Dierking, 2018). Notably, this interest effect is cumulative and requires regular participation to sustain predictive power (Alexander et al., 2012). 

In sum, these studies collectively suggest that informal, family-guided science learning fosters holistic child development—from berry-picking observations (Andrews & Wang, 2017) to structured museum programs (Pagano et al., 2020) and from knowledge exchange to practical engagement—laying a solid cognitive foundation and nurturing lifelong inquiry. Collectively, these findings indicate that out-of-school science activities, through diverse pathways such as knowledge dialogues, hands-on practices, life narratives, and experiential immersion, provide critical momentum for children’s sustained engagement in science learning. They also underscore the importance of constructing rich, continuous, and life-oriented informal science learning environments beyond formal education systems to promote children’s holistic development.

Parent–child science dialogue is characterized by a science-centered focus, mediated through verbal exchange, and oriented toward action-based scientific practices. It employs diverse linguistic frameworks—such as questioning, explanation, and causal discussion—to support children’s science learning and development. Its core objectives are to deepen children’s scientific understanding, enhance their inquiry skills (Acosta et al., 2021; Castañeda et al., 2022; Haden, 2010), and foster cross-contextual transfer abilities (Jant et al., 2014). For instance, an observational study of 75 parent-child dyads found that parents who integrated scientific discussion into play effectively guided children to focus on key material features, enhancing early math skills (Mannweiler et al., 2025). A field study at an aquarium further demonstrated that parental guidance on biological traits and life cycles improved children’s conceptual understanding and increased their accurate use of scientific terminology (Kelly et al., 2020). Notably, science dialogue has been shown to support long-term learning. A museum experiment involving 78 dyads assigned to various intervention groups (cards, objects, cards + objects, control) found that families in the cards group generated more cross-exhibit connections and better recall two weeks later. Children in this group reported an average of 7.33 spontaneous mentions of museum content during follow-up sessions, suggesting improved memory retention through structured dialogue (Jant et al., 2014).

Specifically, questioning guides attention, stimulates thinking, and facilitates knowledge retrieval, thereby influencing children’s observation of scientific phenomena, knowledge construction, and subsequent discussions. Regarding parental questioning, studies found that open-ended questions during shared book reading significantly predicted children’s science knowledge acquisition (Bambha et al., 2024). The effectiveness of questioning also varies by context. In museum settings, closed-ended questions have been shown to strengthen the observation–memory connection and facilitate subsequent discussions. In aquarium settings, closed-ended selective questions (e.g., “Is this a seahorse or a jellyfish?”) were positively correlated with children’s post-visit science talk, with each additional question predicting 2.7 more child-initiated science discussions at home (Ocular et al., 2022). Conversely, in museum environments, closed-ended questions (e.g., “Is this a fossil?”) were unrelated to children’s science discourse (*p* > 0.05) and may even restrict exploration (Leech et al., 2023). These findings suggest that the characteristics of learning environments can influence the effectiveness of dialogic strategies. In settings emphasizing autonomous exploration, dialogic approaches that incorporate intentional pauses and openness are more conducive to stimulating children’s cognitive engagement.

Second, scientific explanations—by providing information, clarifying concepts, establishing connections, and extending knowledge—enhance children’s depth of understanding, memory retention, exploratory behaviors, and interest in science (Callanan et al., 2017; Willard et al., 2019). Explanations that extend beyond the text during book reading were particularly effective in enhancing cognitive and motivational outcomes (Shirefley & Leaper, 2022). Such elaborations were also the strongest predictors of children’s science content recall (Lloyd et al., 2020; Miller-Goldwater et al., 2023b). When caregivers used explanatory talk beyond the text, children retained more information, likely due to the formation of coherent mental representations (Miller-Goldwater et al., 2023a). Experimental evidence further showed that families prompted to explain gear mechanisms engaged in deeper scientific discussion, with children exhibiting higher exploration efficiency (Fender & Crowley, 2007; Willard et al., 2019). Explanation depth and accuracy mattered: parental evidence-based talk positively correlated with children’s exploratory behaviors (Van Schijndel & Raijmakers, 2016), and parental explanation accuracy was positively associated with children’s biology knowledge and verbal intelligence (Mills et al., 2021).

Finally, causal explanations—by providing interpretive frameworks and revealing connections between phenomena—enhance children’s systematic understanding of scientific principles, strengthen their causal reasoning skills, and promote more structured exploratory behaviors (Gin et al., 2025). Observational studies in museum settings revealed that causal language provided cognitive frameworks that facilitated systematic exploration and deeper understanding of circuits and gears (Callanan et al., 2020; Leech, 2024; Legare et al., 2017). Further analysis of 153 dyads found a positive association between the frequency of parental causal talk and children’s causal stance (Booth et al., 2020). In a study of 47 dyads, children were more likely to respond to parental causal questions during scientific inquiry, and these responses were more likely to include science content (Chandler-Campbell et al., 2020).

### 3.4. Family Science Social Capital and Early Childhood Science Learning

This review highlights the positive influence of family social capital on early childhood science learning. A synthesis of 14 relevant studies identified two core components of family social capital: parental science credentials (five studies) and access to science-related social networks (nine studies). These components influence the development of children’s science literacy across four dimensions: knowledge construction, inquiry practice, attitude development, and cultural identification.

Parental science credentials refer to parents’ educational attainment and science-related professional backgrounds, which enhance children’s scientific knowledge, interest, and identity through intergenerational knowledge transmission and role modeling. Several studies included in this review demonstrated that highly educated parents are more capable of facilitating children’s understanding of scientific concepts and supporting their interest in science (Kähler et al., 2020; Suortti et al., 2023; Szechter & Carey, 2009). For instance, observational research conducted in science exhibition settings found that parents with doctoral degrees exhibited greater systematicity when explaining scientific principles, guiding children step-by-step to construct coherent cognitive frameworks (Szechter & Carey, 2009). This approach not only enhanced children’s comprehension of abstract concepts but also stimulated their curiosity and desire for continued exploration. Moreover, parental educational attainment was positively associated with support for children’s science interests. A large-scale survey involving 740 Finnish families found that parents with a bachelor’s degree or higher were more likely to employ diverse strategies to cultivate their children’s interest in science (Suortti et al., 2023). In addition, intergenerational transmission of science-related professions within families showed distinct advantages. Longitudinal research on physical science professionals indicated that having at least one parent employed in a science-related occupation was a critical factor influencing children’s interest and eventual career choice in science (Dabney et al., 2013). A follow-up study involving 4285 doctoral students and scientists in the physical sciences confirmed that parental occupation significantly shaped children’s scientific interests and was a key determinant of their future career trajectory (Dabney et al., 2015).

Science-related social network support refers to families’ connections with individuals possessing science expertise, enhancing children’s science learning through resource sharing, professional input, and cross-context collaboration. Research has shown that support from science professionals enhances not only children’s knowledge acquisition but also their inquiry skills, attitudes toward science, and socio-cultural identity development (Ata-Aktürk & Demircan, 2020; Lloyd et al., 2020). When parents received professional guidance in science education, collaborative engineering activities with their children significantly improved children’s understanding of engineering concepts. These activities cultivated systematic thinking skills such as problem identification, solution design, and iterative improvement. Furthermore, they fostered curiosity, innovation, and collaboration, reinforcing the belief that “engineering is part of daily life” and “anyone can become an engineer” (Ata-Aktürk & Demircan, 2020). Cross-context educational collaborations have further demonstrated that integrated family–school–community ecosystems create immersive science learning environments for preschoolers. For instance, teacher observations confirmed that children who participated in systematized programs demonstrated significantly greater gains in science inquiry skills and interest compared to control groups (Young et al., 2024). In underserved communities, consistent family science engagement was achieved through weekly take-home science books and dialogic reading strategies. Children in intervention groups showed substantial progress in science concept mastery and inquiry skills, with teachers also noting greater learner autonomy (Mantzicopoulos et al., 2013). Moreover, expert support strengthened children’s high-level reasoning by facilitating comparison, prediction, and evaluation processes (Benjamin et al., 2010; Marcus et al., 2018; Vandermaas-Peeler et al., 2017). In conservation tasks, children who received structured science reasoning guidance achieved significantly higher accuracy than control groups (Vandermaas-Peeler et al., 2015). Families also accessed science resources via home science activity kits. An evaluation of five households using inquiry-based kits showed that children remained actively engaged and systematically acquired observational and analytical skills under parental guidance (Strickler-Eppard et al., 2019). These findings indicate that maximizing the value of family science social capital in fostering children’s science learning requires empowering parents as key agents, building supportive networks as safeguards, and promoting collaborative ecosystems as the strategic direction.

## 4. Discussion

Through a systematic literature review grounded in the family science capital framework, this study comprehensively examined how family science capital shapes early childhood science learning. Our findings confirm that family science capital functions as a key determinant of children’s scientific knowledge, skills, attitudes, and socio-cultural identity, operating through three interrelated dimensions—cultural, practice, and social capital—via multi-level and multi-pathway mechanisms. Scholars have engaged in in-depth discussions on these mechanisms, reflecting a strong research focus on cultivating scientific talent and recognizing the critical role of parental science capital. These studies have also substantially informed policymaking, reforms, and practical problem-solving. However, the current research landscape on family science capital and science learning is characterized by a predominance of practice capital studies, a lack of multidimensional longitudinal investigations, and a relatively narrow exploration of cultural capital, limiting a comprehensive understanding of the overall construct and its long-term mechanisms. Moreover, because research between 2007 and 2019 progressed slowly, only 56 relatively recent studies were identified. Notably, the research is highly regionally concentrated, with 84% of studies conducted in North America, primarily in the United States. This concentration suggests that findings may be influenced by specific socio-cultural contexts, restricting their ability to fully represent the complex relationships between family science capital and children’s science learning across diverse countries and cultural settings. Nevertheless, we believe that these studies provide valuable insights for visualizing the broad impacts of family science capital and advancing the analysis of family-level factors influencing early science learning.

Cultural capital primarily influences children’s science learning through the precision of knowledge transmission and the permeation of value-based attitudes, exerting a cumulative effect over time. Children from families with higher levels of parental science literacy and positive science beliefs typically demonstrate greater scientific knowledge and stronger inquiry abilities. Educators should prioritize enhancing parents’ own scientific literacy and fostering positive science beliefs while supporting them in effectively integrating this capital into parent–child interactions.

Practice capital, as the most extensively studied dimension, shapes science learning through contextual immersion, interactive guidance, and reinforcement of learning transfer. These mechanisms operate across multiple levels: (1) Contextuality—embedding science learning in authentic, real-life situations; (2) Interactivity—promoting knowledge sharing and inquiry through parent–child dialogue; (3) Flexibility—adapting activities to children’s needs and interests; and (4) Integrativeness—linking scientific concepts to other disciplines and daily life experiences. This predominance of practice capital research likely reflects its high malleability, low cost, and suitability for empirical measurement, which make it easier to enhance through short-term interventions. However, its benefits are constrained by barriers such as geographic access to informal learning sites, costs, media quality, and parents’ STEM literacy and time investment, underscoring the need for tailored strategies to optimize accessibility and quality.

Social capital contributes by facilitating intergenerational transmission and linking families to broader networks of educational resources. To maximize its value, three strategies are essential: (1) empowering parents through professional training, guidance, and tools; (2) building supportive networks that connect families with external science education resources; and (3) fostering collaborative ecosystems involving families, schools, and communities. This integrated approach is an effective pathway to achieving children’s comprehensive and sustainable development and helps narrow the gap in science learning opportunities across families of differing socioeconomic backgrounds.

Despite contributions from all three dimensions, the current research landscape remains imbalanced. Studies overwhelmingly emphasize practice capital, whereas the roles of family science social networks and value systems are comparatively underexplored. Moreover, research is heavily regionally concentrated, with 84% of studies conducted in North America, primarily in the United States, which raises concerns about the transferability of findings to other cultural contexts. Critically, longitudinal research is scarce, with most studies relying on cross-sectional designs. This lack of longitudinal evidence prevents us from fully understanding the dynamic evolution of family science capital’s influence, particularly whether early advantages translate into sustained academic achievement, deeper science identity formation, and long-term STEM engagement through K–12 and beyond.

Research on science media use and closed-ended questioning, though seemingly contradictory, is in fact complementary. Constructive, co-constructive media engagement—where parents scaffold content into age-appropriate cognitive schemas—creates long-term educational value by fostering children’s science identity. In contrast, passive media consumption (e.g., merely watching cartoons) may boost knowledge retention but lacks depth in abstract concept development and, in low-STEM-literacy households, risks cognitive overload or misinterpretation. Similarly, the effectiveness of closed-ended questions varies by context: in structured environments, they reinforce observation–memory connections, whereas in exploratory settings, open-ended questioning better stimulates cognitive autonomy. These nuances highlight the need for refined strategies in family media use and questioning practices.

## 5. Future Directions and Limitations

To strengthen the evidence base, future studies should address underexplored dimensions (cultural and social capital), adopt innovative methodologies (especially longitudinal designs), and expand cultural diversity by including non-English-language publications and conducting cross-cultural research in Asia, Africa, and other underrepresented regions. Encouraging scholars to conduct systematic reviews in native languages could help integrate diverse findings and build a more inclusive understanding of family science capital. Moreover, meta-analytic and experimental studies are needed to better unpack the contextual mechanisms underlying science media use and questioning strategies.

Policy and practice efforts should prioritize reducing family science capital disparities by promoting equitable resource allocation, optimizing informal science learning ecosystems, and offering low-cost, accessible interventions such as free science materials, financial subsidies, and community-based programs for disadvantaged and immigrant families. Additionally, practical guidance for caregivers should include evidence-based media use guidelines, context-sensitive questioning strategies, and comprehensive frameworks for cultivating family science capital. These efforts should leverage the synergy of media, interaction, and supportive environments to maximize educational impact.

This review is subject to two main limitations. First, while we attempt to interpret the context-dependency underlying seemingly contradictory findings on media use and questioning, more nuanced research designs—including meta-analytic moderation tests and experimental manipulations—are needed to confirm these mechanisms. Second, the review only includes English-language publications, excluding studies published in other languages, which may have constrained the cultural breadth of our analysis.

## 6. Conclusions

This systematic review provides a comprehensive synthesis of how family science capital influences early childhood science learning. Drawing on 56 international empirical studies, it confirms that family science capital serves as a critical lever for promoting children’s science learning by shaping their knowledge acquisition, skill development, attitudinal formation, and socio-cultural identity. Three primary dimensions emerged—cultural, practice, and social capital—yet the roles of cultural and social capital remain underexplored. Importantly, family science capital comprises multiple interacting components whose configurations produce diverse outcomes, underscoring the need for examining these interrelationships more closely through innovative research methods. Moreover, equitable access to high-quality science education is a prerequisite for achieving educational equity. Enhancing family science capital, particularly for disadvantaged families with lower levels of inherited capital, must be a key policy priority. Building collaborative ecosystems and fostering interdisciplinary, cross-cultural, and intersectoral partnerships can help transform theoretical calls for action into practical strategies. Such efforts are crucial for mitigating the constraints of limited family capital and promoting sustainable improvements in children’s early science learning.

## Appendix A. Characteristics of Included Studies

**Included Study**	**Year**	**Country**	**Type of Study**	**Methodology**	**Sample**	**Family Science Capital Dimension**
(Andrews & Wang, 2017)	2017	the United States	Empirical	Qualitative research	A 7-year-old girl and her mother	Participation in out-of-school science activities
(Miller-Goldwater et al., 2023a)	2023	the United States	Empirical	Mixed research	38 children and their caregiver	Science talk with children
(Leech, 2024)	2024	the United States	Empirical	Quantitative research	A total of 70 parents with a 4- or 5-year-old child	Science talk with children
(Young et al., 2024)	2024	the United States	Empirical	Mixed research	29 total educators and 24 Focus Families	Science-linked social networks
(Suortti et al., 2023)	2023	Finland	Empirical	Quantitative research	740 parents	Parental education level
(Mantzicopoulos et al., 2013)	2013	the United States	Empirical	Mixed research	243 kindergarten children	Science-linked social networks
(Bae et al., 2023)	2023	the United States	Empirical	Quantitative research	153 children, age range from 3 to 8 years old	Participation in out-of-school science activities
(Paños & Ruiz-Gallardo, 2020)	2020	Spain	Empirical	Quantitative research	62 children, aged from 5 to 8 years old and their parents	Participation in out-of-school science activities
(Junge et al., 2021)	2021	Germany	Empirical	Quantitative research	257 5-year-old children and their parents	Participation in out-of-school science activities
(Shirefley & Leaper, 2022)	2022	the United States	Empirical	Mixed research	50 families and their children aged between 4 and 7 years old	Science talk with children
(Lloyd et al., 2020)	2020	the United Kingdom	Empirical	Qualitative research	26 children aged under 5 and 19 parents	Science-linked social networks
(Mills et al., 2021)	2021	the United States	Empirical	Quantitative research	148 parent–child	Science talk with children
(Pagano et al., 2020)	2020	the United States	Empirical	Quantitative research	61 6- to 8-year-old children and their families	Participation in out-of-school science activities
(Haden, 2010)	2010		Review			
(Strickler-Eppard et al., 2019)	2019	the United States	Empirical	Qualitative research	Five households, each consisting of at least one adult and 2–3 children, one of whom is the target age (4–8 years).	Science-linked social networks
(Legare et al., 2017)	2017		Review			
(Polinsky et al., 2023)	2023	the United States	Empirical	Mixed research	243 families with children aged 6–11 years	Science talk with children
(Haden et al., 2023)	2023		Review			Participation in out-of-school science activities
(Leech et al., 2023)	2023	the United States	Empirical	Qualitative research	49 children aged 4–6 years and their parents	Science talk with children
(Castañeda et al., 2022)	2022	the United States	Empirical	Qualitative research	243 families with children aged 3–5 years	Science talk with children
(Ocular et al., 2022)	2022	the United States	Empirical	Quantitative research	38 children and their parents	Science talk with children
(Acosta et al., 2021)	2021	the United States	Empirical	Quantitative research	111 families with children aged 5–10 years	Science talk with children
(Chandler-Campbell et al., 2020)	2020	the United States	Empirical	Quantitative research	47 parent–child and the children aged 4–6 years	Science talk with children
(Chandler-Campbell et al., 2020)	2020	the United States	Empirical	Quantitative research	60 parent–child and the children aged 4–5 years	Science talk with children
(Callanan et al., 2020)	2020	the United States	Empirical	Qualitative research	111 families with children aged 3–4 years	Science talk with children
(Pattison & Dierking, 2018)	2018	the United States	Empirical	Qualitative research	7 mothers with 4-year-old daughters	Participation in out-of-school science activities
(Marcus et al., 2018)	2018	the United States	Empirical	Quantitative research	64 4- to 8-year-old children and their parents	Science-linked social networks
(Eberbach & Crowley, 2017)	2017	the United States	Empirical	Quantitative research	79 families with children aged 6–10 years	Scientific literacy
(Jant et al., 2014)	2014	the United States	Empirical	Quantitative research	78 families with children aged 4–6 years	Science talk with children
(Benjamin et al., 2010)	2010	the United States	Empirical	Quantitative research	121 4- to 8-year-old children and their caregiver	Science-linked social networks
(Szechter & Carey, 2009)	2009	the United States	Empirical	Quantitative research	60 parent–child and the children aged 5.6–14.9 years	Parental education level
(Dou et al., 2019)	2019	the United States	Empirical	Quantitative research	15,847 students at 27 colleges and universities	Participation in out-of-school science activities
(Callanan et al., 2017)	2017	the United States	Empirical	Quantitative research	83 parent–child and the children aged 3–11 years	Science talk with children
(Alexander et al., 2012)	2012	the United States	Empirical	Quantitative research	192 4- to 7-year-old children and their parents	Participation in out-of-school science activities
(Fender & Crowley, 2007)	2007	the United States	Empirical	Quantitative research	children aged 3–8 years	Science talk with children
(Dabney et al., 2015)	2015	the United States	Empirical	Quantitative research	4285 chemistry and physics doctoral students, scientists, and individuals with other doctoral degrees in physical science.	Parents’ occupational background
(Dabney et al., 2013)	2013	the United States	Empirical	Quantitative research	4285 chemistry and physics doctoral students, scientists, and individuals with other doctoral degrees in physical science.	Parents’ occupational background
(Westerberg et al., 2022)	2022	the United States	Empirical	Quantitative research	125 families with children aged 3 to 5 years	Participation in out-of-school science activities
(Booth et al., 2020)	2020	the United States	Empirical	Quantitative research	153 3-year-old children and their parents	Science talk with children
(Kähler et al., 2020)	2020	Spain	Empirical	Quantitative research	2937 children in kindergarten	Parental education level
(Willard et al., 2019)	2019	the United States	Empirical	Quantitative research	65 parent–child and the children aged 4–6 years	Science talk with children
(Vandermaas-Peeler et al., 2017)	2017	the United States	Empirical	Quantitative research	32 families with children aged 4 to 5 years	Science-linked social networks
(Vandermaas-Peeler et al., 2015)	2015	the United States	Empirical	Quantitative research	25 families with children with a mean age of 67 months	Science-linked social networks
(Van Schijndel & Raijmakers, 2016)	2015	Netherlands	Empirical	Quantitative research	89 parent–child and the children aged 4–6 years	Science talk with children
(Pattison et al., 2020)	2020	the United States	Empirical	Qualitative research	Kindergarten children with their parents	Scientific belief
(Kelly et al., 2020)	2020	the United States	Empirical	Quantitative research	22 children aged 3–8 years and their parents	Science talk with children
(Alexandre et al., 2022)	2022		Review			
(Dominke et al., 2025)	2025	Germany	Empirical	Quantitative research	61 parent–child and the children aged 4–7 years	Participation in out-of-school science activities
(Miller-Goldwater et al., 2023b)	2023	the United States	Empirical	Quantitative research	38 children and their caregiver	Science talk with children
(Salvatierra & Cabello, 2022)	2022		Review			
(Kontkanen et al., 2024)	2024		Review			
(Bambha et al., 2024)	2024	the United States	Empirical	Quantitative research	173 parents and their children	Science talk with children
(Sheehan et al., 2018)	2018	the United States	Empirical	Quantitative research	96 parents of preschool-aged children	The use of scientific media
(Gin et al., 2025)	2025	the United States	Empirical	Quantitative research	68 children aged 4–7 years	Science talk with children
(Ata-Aktürk & Demircan, 2020)	2020	Turkey	Empirical	Qualitative research	Two ECE teachers, five children, and five parents	Science-linked networks
(Mannweiler et al., 2025)	2025	the United States	Empirical	Quantitative research	75 kindergarten children with their parents	Science talk with children

## Appendix B. Keywords

**Table:**

Keywords	Synonyms
Set A: science capital	scientific capital
	family science capital
	parental science capital
	household science capital
	science-related cultural capital
	scientific literacy
	science beliefs
	science career value
	science media consumption
	science television
	science books
	popular science articles
	informal science learning
	science social capital
	science-related social ties
	science network
	science discussion frequency
	science reference group
Set B: early science learning	early science learning
	early childhood science
	preschool science
	young children science
	science interest
	science knowledge
	scientific reasoning
	science reflection
	science identity
	science engagement
	science practice
	STEM identity
	scientific thinking
	science attitude
Set C: home environment	family
	parent
	home environment
	caregiver
	household
	maternal
	paternal
Set D: Age group	early childhood
	preschool
	Kindergarten
	young child
	nursery school
	0–8 years
	pre-primary

Search1: Set A* AND Set B * AND Set C*AND Set D*. Search2: Set A* AND Set B * AND Set C*AND Set D*. Search3: Set A* AND Set B * AND Set C*AND Set D*. Search4: Set A* AND Set B * AND Set C*AND Set D*.

## Appendix C. Thematic Framework for Family Science Capital

**Final Theme**	**Subtheme**	**Representative Initial Codes**	**Definition/Description**	**Logic for Code Generation**	**Basis for Subtheme Clustering**
Family Cultural Science Capital	Scientific Literacy	Mastery of Scientific Knowledge, Use of Disciplinary Terminology	The extent to which family members possess scientific knowledge, methods, and principles.	Directly extracted from raw texts mentioning ‘scientific literacy,’ ‘parents’ mastery of scientific knowledge,’ and ‘parents’ use of scientific knowledge.	Merged all knowledge-related codes (e.g., ‘use of terminology,’ ‘conceptual associations’), focusing on cognitive foundations.
	Science Dispositions and Preferences	Recognition of the Value of Science, Emotional Orientation toward Science, Importance Placed on Science	Family members’ recognition of the social value of science, emotional attitudes, and the level of importance attached to science.	Captured from raw texts reflecting parental attitudes (e.g., ‘science is important’).	Combined scientific value judgments and emotional expressions (interests and preferences) to reflect the attitudinal dimension.
	Science Labor Market Conversion	Recognition of the Value of Science Careers, Transmission of Career Knowledge	The extent to which family members understand the types, content, and social value of science-related (STEM) professions.	Extracted from raw texts indicating parents’ attitudes toward science careers (e.g., ‘understanding of types and content of science careers,’ ‘awareness of the labor market value of science careers’).	Combined recognition of the social value of science careers with descriptions of their types, content, and value.
Family Practice Science Capital	Science Media Consumption	Frequency of Watching Science Programs, Digital Media Interaction Frequency	The frequency and depth with which family members engage with science content via media such as television and the internet.	Directly extracted from raw texts referring to ‘science media, resources, digital tools,’ and ‘usage frequency of digital resources.’	Combined codes on using science media, resources, and digital tools.
	Participation in Out-of-School Science Activities	Visits to Science Venues, Family Experiments and Exploration, Reading Science Books	Participation in science exploration activities in informal settings (e.g., museums, family-based activities).	Analyzed and directly extracted from texts mentioning ‘family science activities,’ ‘visits to science venues,’ and ‘reading science books.’	Integrated content focusing on participation in out-of-school activities, especially those emphasizing contextual experiences.
	Science Dialogue	Questioning, Scientific Explanations, Causal Discussions	Dialogue patterns in which family members engage in questioning, explanations, and causal reasoning around scientific phenomena.	Classified based on question type (open-ended questions with interrogatives vs. closed questions with yes/no judgments). Identified parent explanations and causal reasoning using words like ‘because/mechanism.’	Unified dialogue strategies (questioning, explaining, causal discussions) as dialogic behaviors, distinguishing them from media activities or venue-based practices.
Family Social Science Capital	Science Credentials	Parental Educational Background, Parental Science-Related Occupations	The educational level of family members and their professional backgrounds in science-related fields.	Identified from raw texts referring to ‘parental education level’ and ‘science-related occupations.’	Combined education level and science-related career background as the credentials dimension.
	Science Network Support	Collaboration with Professional Institutions, Home–School Science Collaboration, Use of Toolkits	Access to scientific resources and support through schools, communities, experts, and other external networks.	Captured key collaborative contexts (e.g., ‘teacher guidance for parents,’ ‘family participation in school science days,’ ‘use of family toolkits,’ ‘parent training’).	Merged external resource engagement behaviors (institutional collaboration, expert guidance, toolkit use), distinguishing them from internally generated family capital.

## Appendix D. Examples of Thematic Coding Process

**Raw Data Excerpt (Anonymized)**	**Initial Codes**	**Conflict Handling**	**Subtheme**	**Theme**	**Code Generation Rationale**
“Parents with high scientific literacy used terms like ‘stamen’ and ‘pollen transfer’ more frequently while observing flowers in a botanical garden and guided children to understand the adhesion mechanism of pollen to bees’ leg hair.”	Mastery of Scientific Knowledge; Use of Disciplinary Terminology	—	Scientific Literacy	Family Cultural Science Capital	The original codes (“use of terminology”) reflect understanding of scientific principles, representing cognitive foundations.
“Parents’ use of closed-ended questions (e.g., ‘Is this a fossil?’) in a museum was not significantly associated with children’s science-related talk.”	Closed-Ended Questioning	—	Science Dialogue	Family Practice Science Capital	Generated directly based on sentence structure (yes/no or true/false judgment).
“Parents with doctoral degrees were better able to help their children establish a complete cognitive framework for circuit principles.”	1. Parental Educational Background; 2. Scientific Literacy	Marked as a conflicting case and, after analysis, categorized under “Science Credentials.”	Science Credentials	Family Social Science Capital	Conflicting statements were flagged and discussed. Since the focus was on parental education level, it was categorized under science credentials.
